# Development and validation of a Chinese version of a questionnaire to evaluate the knowledge, attitude, and practice about breast cancer screening among financial female workers in Taiwan

**DOI:** 10.3389/fonc.2025.1559622

**Published:** 2025-06-26

**Authors:** Jia-Yi Lin, Ching-I Hung, Tsai-Chung Li, Ta-Yuan Chang

**Affiliations:** ^1^ Department of Public Health, College of Public Health, China Medical University, Taichung, Taiwan; ^2^ Department of Occupational Safety and Health, College of Public Health, China Medical University, Taichung, Taiwan; ^3^ Institute of Labor, Occupational Safety and Health, Ministry of Labor, New Taipei, Taiwan; ^4^ Institute of Environmental and Occupational Health Sciences, National Taiwan University, Taipei, Taiwan

**Keywords:** cancer screening questionnaire, construct validity, content relevance, cross-sectional study, domain coverage, internal consistency

## Abstract

**Introduction:**

This study aimed to develop a Chinese version of a questionnaire to assess the knowledge, attitude, and practice of breast cancer screening among female workers in the financial industry and to evaluate its reliability and validity.

**Methods:**

An item pool relevant to knowledge of and attitudes toward breast cancer screening was generated, and 16 experts assessed the validity of the instrument’s content relevance and domain coverage. We conducted a cross-sectional study of 1,511 women working in the financial industry in Taiwan. The questionnaire’s construct validity was assessed using correlations between the items and other scales to evaluate knowledge of and attitudes toward breast cancer screening. The internal consistency was evaluated using Cronbach’s α values.

**Results:**

Positive and negative attitudes toward mammography and attitudes toward reasons for not receiving a mammography accounted for 68.3%, 10.3%, and 20.9% of total variance, respectively. The Cronbach’s α coefficients for knowledge of and attitudes toward breast cancer screening were 0.37 and 0.91, respectively. One interquartile range (IQR) increase in the total scores on the breast cancer attitudes toward mammography (6 points; adjusted odds ratio [AOR]: 1.47, 95% confidence interval [CI]: 1.24-1.75) and the total scores on the reasons for not receiving a mammography (8 points; AOR: 1.79; 95% CI: 1.46-2.20) were significantly associated with the practice of having ever received a mammography. Both scores showed significant exposure-response associations.

**Conclusion:**

Our findings indicate that the Chinese version of the questionnaire used to evaluate attitudes and practices toward breast cancer screening among female workers in the financial industry demonstrated good construct validity and internal consistency.

## 1 Introduction

Breast cancer is the most diagnosed cancer among women worldwide. The International Agency for Research on Cancer (IARC) of the World Health Organization reported that new breast cancer cases will surpass lung cancer cases in 2020 (1). Breast cancer ranks first among females in Taiwan, accounting for 12.3% of all cancer cases in 2020 (2). A previous study found that all-occupation female workers had an age-standardized incidence of breast cancer that increased from 59.99 to 87.17 per 100,000 person-years from 2010 to 2018 (3).

Many occupational studies have demonstrated a significantly higher incidence of breast cancer among female workers than women in the general population, such as flight attendants (4), electronics workers (5), professional and managerial workers (6), and women employed in the financial, insurance, and real estate industries (7). In addition to specific exposures (such as ionizing radiation and solvents) in different industries, female workers have multiple roles in balancing occupational and household responsibilities (8), which could increase stress in their daily lives and decrease their willingness and behavior to participate in breast cancer screening.

Previous studies have developed knowledge, attitude, and practice (KAP) questionnaires for breast cancer (9–11), and Andersen’s behavioral models have been established continuously in recent years, focusing on individual health practices and status (12–14). In the context of developing new scales, these models underscore the importance of evaluating both the structural and perceptual elements that drive health-service utilization. However, these tools may not be applicable globally because of differences in national culture, governmental policies, educational levels, and occupations. Although universal scales are valuable, local adaptations are necessary to ensure their cultural relevance and accuracy in reflecting the health practices and beliefs of the local population.

Breast cancer screening can help improve public health and cancer prevention through early detection. Clarifying the reasons for women’s increased willingness to undergo breast cancer screening is critical, as it can improve participation rates. However, the KAP related to breast cancer screening among female workers in the financial industry remains unclear, particularly because of unique occupational, social, and psychological factors. Understanding these factors is crucial for developing effective health interventions and policies to improve breast cancer screening rates among female workers. Thus, this study aimed to develop a Chinese version of the questionnaire that evaluates the KAP for breast cancer screening among female workers in the financial industry in Taiwan. It also sought to investigate the potential association between their knowledge, attitudes, and mammography practices.

## 2 Materials and methods

### 2.1 Study design and subjects

This cross-sectional study recruited 1,511 women working in Taiwan’s financial industry using two-stage sampling from June to November 2022. The first stage utilized stratified sampling of financial institutions by scale (i.e., ≥ 6000, 3000–5999, and < 3000 employees) and type (i.e., public or private). Based on the distribution of the total population in the financial industry from the National Labor Insurance Database, the second stage adopted quota sampling of employees according to the total number of employees in Northern, Central, Southern, and Eastern regions, comprising 75.0%, 12.0%, 8.0%, and 5.0%, respectively. We contacted the occupational health nurses at each financial institution to recruit participants and increase their participation rates. All occupational health nurses communicated with each other before recruitment.

The study protocol was approved by the Central Regional Research Ethics Committee of China Medical University and Hospital, Taichung, Taiwan (Protocol Number: CMUH111-REC3-078). All participants provided written informed consent before participation.

### 2.2 Measurements

#### 2.2.1 Development of a Chinese version of the questionnaire

The questionnaire was developed in two steps: item pool and questionnaire development and a readability test and reliability evaluation (**Supplementary Figure S1**). The first step was to define the construct concepts of breast cancer screening and generate items to assess the questionnaire’s content validity. A total of 58 items were generated from a review of the existing literature on breast cancer screening, including women in Taiwan (15, 16), Australia (17), Iran (18), and female workers in Taiwan’s medical institutions (19).

#### 2.2.2 Content and construct validity

After generating an item pool, a panel of 16 experts assessed the content validity of the 58 items using the following equation (Equation 1) to evaluate the content-validity ratio (CVR) (20):


(1) CVR=Ne−N2N2

where Ne is the number of experts who judged the item as important for a specific topic in the assessment tool and N is the total number of experts who judged the item. The CVR can be used to measure the difference in percentages between the actual and expected number of experts judging an item as important. The content-validity results are shown in **Supplementary Tables S1–S4**.

Second, the construct validity evaluation determined the degree to which the content of the measurement tool had the characteristics to be measured. This study examined item- and scale-level convergent and discriminant validity using Pearson’s coefficients to estimate the strength of the correlation. The item-level convergent validity estimates the correlation between each item and the total score of the scale containing it. Convergent validity was assumed when the correlation coefficient was greater than 0.40 (21), which is an assumption for evaluating a Likert scale. The item-level discriminant validity estimated whether the correlation between each item and its scale was higher than that between the other scales. If it was significantly higher, it demonstrated discriminant validity, which has no fixed critical value for this validity. Additionally, a factor analysis of the item coefficients was conducted to identify the underlying constructs that explain the pattern of correlations within a set of observed variables. A coefficient value greater than 0.40 represents the item suiting the belonging factor.

#### 2.2.3 Questionnaire pretest

To generate the final version, which included 38 items after the first step, the instrument was shortened by excluding items with similar meanings and no variation. To clarify and simplify the text wording, the second step included a pre-test to assess the readability of the questionnaire with 20 participants from another eligible financial institution. This pre-test was performed to understand the meaning of the content, independence of options, and whether the instrument could achieve our study’s purpose. To complete the final version of the questionnaire, participants were invited to provide feedback to ensure that each question produced sufficient answers. Finally, 38 items in the questionnaire have been reduced to 32 ones as the final version, which includes 15 questions for knowledge of and 17 ones for attitudes toward breast cancer screening.

#### 2.2.4 Reliability test

A pilot study that included 64 volunteers from another financial institution was conducted to evaluate the test-retest reliability and internal consistency. The same questionnaire was administered to the 64 volunteers twice, with a two-week time interval to avoid testing effects. To prevent dependence on the survey, the results of the second test were not included in the valid questionnaires used for data analysis. In addition, the Cronbach’s α coefficient of each scale (i.e., reliability of internal consistency) was evaluated (22).

### 2.3 Statistical analysis

Descriptive statistics were presented for the sociodemographic factors of the 1,511 participants. Pearson’s correlation coefficient was used to assess the correlation between breast cancer screening knowledge and attitudes with item- and scale-level convergent and discriminant validity. Independent t-tests and one-way analysis of variance (ANOVA) tests were used to explore the differences between sociodemographic factors and breast cancer screening knowledge, attitudes, and practice. Cronbach’s α coefficient was applied to assess the internal consistency, and an α ≥0.80 was considered to indicate good internal consistency (22).

Logistic regression models were used to explore the associations between sociodemographic factors and participation in the mammography, which were reported using odds ratios (OR) and 95% confidence intervals (CI). To predict the likelihood of engaging in screening practices, the predictive validity of the psychometric properties was examined to evaluate variations in the knowledge and attitude scales. All psychometric scale scores were standardized before inclusion in the regression models. According to the results of the t-tests and ANOVAs, those with p-values greater than 0.25 were included in the multivariate logistic regression for data analysis (23, 24). This process eliminated the variables with the largest p-values related to the dependent variable, one at a time, by manual inspection. After repeating this process, the variables that reached statistical significance with the dependent variable were included in the final model. All analyses were performed using the SAS standard package for Windows version 9.4 (SAS Institute Inc., Cary, North Carolina, USA).

## 3 Results

### 3.1 Development results of survey

**Table 1** presents the sociodemographic characteristics of participants in the financial industry. The mean age among participants was 42.6 ± 10.4 years old, with those over 40 years old representing 64.8% of the participants. More than half participants were from the Northern region. A small number of patients with breast cancer (n=22; 1.5%) were diagnosed by a physician. The distribution of the items on breast cancer screening knowledge, attitude, and practice is shown in **Supplementary Tables S5-S7**. Of all participants, 98.2% (n = 1,484) reported that they had undergone X-ray mammography (**Supplementary Table S7**). Among them, 49.1% (n = 728) indicated that they had undergone mammography. Missing data (1.8%) were excluded from the analysis.

**Table 1:** Sociodemographic characteristics of participants based on the Chinese version of the questionnaire for breast cancer screening.

Variables	Subjects	
	N (%)	Mean ± SD
Age (years)		42.6 ± 10.4
<40	532 (35.2)	
≥40	979 (64.8)	
Body mass index (kg/m^2^)		21.9 ± 3.0
Living area	14 (0.9)^a^	
Northern	845 (55.9)	
Western	309 (20.5)	
Southern	328 (21.7)	
Eastern	15 (1.0)	
Education level	4 (0.3)^a^	
Senior high school education or below	105 (7.0)	
College or University degree, or above	1,402 (92.8)	
Average monthly household income (NTD)	11(0.7)^a^	
<100 thousand	942 (62.5)	
≥100 thousand	558 (36.9)	
Marital status	1 (0.1)^a^	
Single	498 (33.0)	
Married	959 (63.5)	
Divorced	46 (3.0)	
Widowed	7 (0.5)	
Cigarette smoking, current	8 (0.5)	
Alcohol consumption, current	76 (5.0)	
Size of institutions	0 (0.0)	
Small	398 (26.3)	
Medium	205 (13.6)	
Large	908 (60.1)	
Type of position	3 (0.2)^a^	
Clerk	904 (59.8)	
Supervisor	276 (18.3)	
Others	328 (21.7)	
Current menstrual status, yes	1,176 (77.8)	
Breast cancer related cases		
Diagnosed with breast cancer by a physician	22 (1.5)	
Female relative with breast cancer	264 (17.5)	

a Missing data for each variables.

**Table 2** presents the factor structure of breast cancer screening attitudes. All the factors had coefficients greater than 0.40. The three-factor model accounted for 99.5% of the total variance. The first factor included items related to “Positive Attitudes toward Mammography”, the second related to “Attitudes Regarding the Reasons for Not Receiving a Mammography”, and the third related to “Negative Attitudes toward Mammography”. The three factors accounted for 68.3%, 20.9%, and 10.3% of the total variance, respectively.

**Table 2:** Coefficients of items for breast cancer screening attitude using principal components method with varimax rotation for evaluating the constructs (N=1,511).

Items	Factor 1 Positive attitudes towards mammography	Factor 2 Attitude on the reasons for not participating in mammogram	Factor 3 Negative attitudes towards mammography	Common Factor Variance Estimates
BCA1: A mammogram is not necessary if I believe my breasts are healthy.	0.22	0.28	0.80*	0.77
BCA2: A mammogram is not necessary because I live a healthy life (i.e., regular exercise, healthy diet).	0.25	0.27	0.83*	0.82
BCA3: I have the ability to check up my breasts, so I do not need to participate in the mammogram.	0.28	0.27	0.67*	0.61
BCA4: It is safe for me to have a mammogram.	0.77*	0.21	0.11	0.64
BCA5: It is necessary for me to have a mammogram.	0.77*	0.25	0.24	0.72
BCA6: Regular mammograms keep me updated on the health of my breasts.	0.89*	0.16	0.18	0.85
BCA7: Regular mammograms provide medical information about breast cancer.	0.83*	0.13	0.12	0.73
BCA8: Even if every breast self-exam is normal, I should keep getting mammograms.	0.76*	0.19	0.31	0.71
BCA9: I believe that participating in a mammogram will determine abnormalities early.	0.82*	0.13	0.15	0.72
BCA10: I am worried that having a mammogram will hurt my breasts.	0.34	0.45*	0.20	0.36
BCA11: I was uncomfortable with the idea of exposing my breasts during the mammogram.	0.18	0.62*	0.17	0.45
BCA12: Mammograms are painful, so I do not want to have them.	0.17	0.61*	0.17	0.43
BCA13: I am too busy at work, so I do not have time for mammograms.	0.90	0.71*	0.12	0.53
BCA14: Lack of transportation would keep me from having a mammogram.	0.10	0.76*	0.11	0.61
BCA15: Having breast cancer would affect my life, so I do not want to have a mammogram.	0.23	0.52*	0.29	0.41
BCA16: The cost is too high, which discourages me from getting a mammogram.	0.13	0.64*	0.19	0.47
BCA17: There are no health facilities near my home to provide mammograms, so I do not want to have a mammogram.	0.12	0.75*	0.13	0.60
Accounting for total variance	68.3%	20.9%	10.3%	−

BCA, questions on breast cancer attitude; Reliability evaluation: Cronbach’s α coefficient: 0.78-0.93; *Convergent validity: A correlation ≥0.40.

### 3.2 Validation results of survey

Pearson’s correlation coefficients were calculated to estimate the strength of the relationship between each item and the total score on the scale to which it belonged. The variation in the standard deviations of items belonging to knowledge and attitude scales were 0.11 to 1.18, while the variation of item-scale correlations within scales ranged from -0.02 to 0.68 (**Table 3**). All 17 item-scale correlations for attitudes were satisfied by the prior convergent validity criterion (i.e., ≥0.40), and the correlations for discriminant validity of 17 items were higher than 87.5% compared with other scales assessing attitudes.

**Table 3:** The characteristics of scale between breast cancer screening knowledge and attitude.

Scales	Item S.D. (range)	Correlations of items with its scale (range)	Correlations of items with other scale (range)	Convergent validity	Discriminant validity
Knowledge	0.11−0.47	-0.02−0.26	0.32−0.41	0.0% (0/15)	33.3% (5/15)^a^
Knowledge on breast cancer	0.24−0.47	-0.03−0.25	0.15−0.30	0.0% (0/8)	25.0% (2/8)^b^
Knowledge on breast cancer screening	0.11−0.43	-0.01−0.32	0.21−0.42	0.0% (0/7)	42.9% (3/7)^b^
Attitude	0.62−1.18	0.56−0.68	0.91−0.91	100.0% (17/17)	94.1% (16/17)^a^
Attitude on doing a mammography	0.62−0.94	0.62−0.80	0.90−0.91	100.0% (9/9)	100.0% (9/9)^b^
Attitude on the reasons for not participating in mammogram	0.73−1.18	0.51−0.71	0.84−0.86	100.0% (8/8)	87.5% (7/8)^b^

^a^Discriminant validity between scales; ^b^ Discriminant validity of sublevels under the same scale.

The **Supplementary Table S8** shows that Cronbach’s α coefficients of the scales for knowledge and attitude were 0.25 to 0.37 and 0.87 to 0.91, respectively. The results of the reliability test on breast cancer showed that 60.0% of the knowledge questions had a kappa value greater than 0.40 (25) and 100.0% of the practice section provided good reliability (**Supplementary Table S9**). Differential analysis of the practice of participating in breast cancer screening is shown in **Supplementary Table S10**. Additionally, higher levels of knowledge and attitude scales were significantly associated with mammography practices.

### 3.3 Practice results of survey

**Figure 1:**
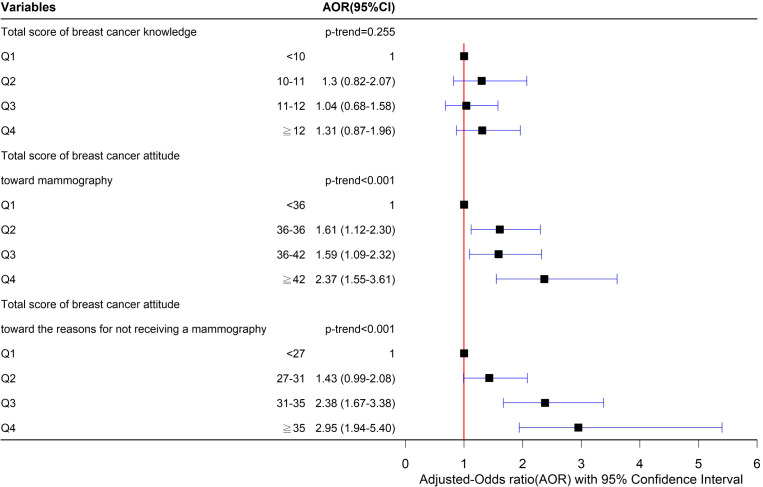
Associations between ever receiving the mammogram and the total score of knowledge and attitude.

The associations between sociodemographic factors and the practice of ever receiving a mammography are shown in **Supplementary Table S11**. One interquartile range (IQR) increase in the total score of breast cancer attitudes regarding receiving a mammography (6 points, adjusted odds ratio [AOR]=1.47, 95% CI: 1.24-1.75) and in the total score of breast cancer attitudes toward the reasons for not receiving a mammography (8 points, AOR=1.79, 95% CI: 1.46-2.20) makes the participant more likely to receive a mammogram. We further estimated the associations between the practice of ever receiving a mammography and the total score of breast cancer knowledge and attitudes by quartile (**Figure 1**). Significantly increasing trends in the total score of breast cancer attitudes toward receiving a mammography (AOR=1.47, 95% CI: 1.24-1.75, p<0.001) and in that toward the reasons for not receiving a mammography (AOR=1.79, 95% CI: 1.46-2.20, p<0.001) were observed in this study.

We also analyzed the associations between sociodemographic factors and the practice of self-checking (**Supplementary Table S12**) and ultrasound examinations (**Supplementary Table S13**). One IQR increase in the total score for breast cancer knowledge (2 points, AOR=1.37, 95% CI: 1.15-1.64) and in the total score for breast cancer attitudes toward the reasons for not receiving a mammography (8 points, AOR=1.43, 95% CI: 1.16-1.77) were significantly associated with the practice of having ever conducted a breast self-check. Furthermore, one IQR increase in the total score for breast cancer attitudes toward receiving a mammography (6 points, AOR=1.45, 95% CI: 1.22-1.72) and in that toward the reasons for not receiving a mammography (8 points, AOR=1.62, 95% CI: 1.31-2.01) were significantly associated with the practice of having ever received an ultrasound examination.

## 4 Discussion

### 4.1 Development and validation of survey

This study demonstrates the development of an important assessment tool using a purposeful, contextual, and logical approach to measure KAP related to breast cancer screening among female workers in the financial industry in Taiwan. For the questionnaire’s content validity, 96.6% (i.e., 56/58) of the original items were found to be important questions (i.e., CVR ≥0.50). It is essential to ensure that every item appropriately corresponds to the specific scale (20, 26). Regarding construct validity, the knowledge items did not demonstrate convergent validity (i.e., the knowledge items did not successfully capture and explain the variance in the scale through the constructs) and only reached 33.3% (i.e., 5/15) discriminant validity (i.e., the knowledge item reached one-third of the variance within the other scale). This weak validity could be attributed to the broader spectrum of breast cancer screening knowledge encompassed in the questionnaire’s content. Another possible reason for this could be the broad and diverse nature of the knowledge questions, which covered various aspects of breast cancer and its screening, thereby reducing inter-item correlations. However, the attitude items exhibited 100.0% convergence (i.e., all items in the questionnaire consistently measured the intended constructs) and 94.1% (i.e., 16/17) discriminant validity, confirming that each attitude item effectively contributed to measuring the specific and designed aspects of the construct. Furthermore, the percentage of kappa values exceeding 0.40, indicating moderate agreement, was 60.0% (i.e., 9/15) for knowledge items and 100.0% (i.e., 3/3) for practice items. The moderate-to-high agreement in the knowledge and practice items indicates the reliability of the survey instrument and bolsters the credibility of our findings.

In this study, “negative attitudes” were conceptualized as cognitive beliefs that downplay the perceived necessity of mammographic screening. These attitudes are rooted in an individual’s confidence in personal health practices, such as maintaining a healthy lifestyle, performing breast self-examinations, or the belief that screening is unnecessary in the absence of symptoms. Rather than being driven primarily by emotional discomfort or fear, these attitudes reflect a cognitive evaluation of the need for screening, which is often shaped by personal beliefs, behaviors, or misconceptions about cancer risk.

In contrast, the subscale “reasons for not participating” refers to situational or practical barriers that directly affect an individual’s ability or willingness to attend screening, such as time constraints, fear of pain, and embarrassment. While certain items (e.g., embarrassment or fear of pain) could theoretically fall under both constructs, item classification was guided by both the theoretical rationale and empirical results of the factor analysis. This dual approach allowed us to distinguish between underlying belief-based attitudes and context-specific reasons for non-participation, thereby ensuring that the factor structure remained conceptually meaningful and psychometrically sound.

### 4.2 Practice of results of survey

Our study indicated that participants’ attitudes were significantly associated with the practice of participating in mammography, including their attitudes toward mammography and their reasons for not participating. Participants with more positive attitudes (i.e., those with higher total scores for attitude items) were more likely to receive a mammography. A previous study conducted in India revealed that women with adequate awareness of breast cancer (32.0%) and those willing to receive more information about breast cancer (27.0%) were more likely to undergo breast cancer examinations (27). These beliefs may motivate women to actively participate in a mammography. In terms of attitudes toward the reasons for not receiving a mammography, a higher total score on these items indicated a stronger disagreement with the notion that these factors impede participation in screening. These women exhibited positive attitudes toward statements, such as “Having breast cancer would affect my life, so I do not want to have a mammogram”, “I am worried that having a mammogram will hurt my breasts”, and “I was uncomfortable with the idea of exposing my breasts during the mammogram”. Thus, these concerns were not significant factors for women, suggesting that raising positive attitudes could improve mammography practices.

However, other important reasons were observed for women who elect to not receiving a mammogram. Participants agreed that being too busy at work (coefficient = 0.71 for BAC 13), lack of transportation (coefficient = 0.76 for BAC 14), and experiencing pain during mammography (coefficient = 0.61 for BAC 12) (**Table 2**) were reasons for not receiving a mammogram. These findings suggest that practical barriers and physical discomfort are critical factors for breast cancer screening. Additionally, fear of discomfort may serve as a psychological deterrent. A Saudi Arabian study found that being busy with a lack of time, previous bad experiences with healthcare providers, stigma following the diagnosis of cancer, and shame in uncovering one’s breasts were significant barriers to participating in breast cancer screening (28). Thus, incorporating breast cancer screening into employees’ physical examinations, deploying mammography vehicles near the company to provide screening services, and providing clear information about the mammography process should be considered to increase willingness to screen.

Our findings regarding the knowledge items revealed that participants achieved a high score (i.e., 73.9%, 11.1/15) on the questionnaire. Most participants were knowledgeable about breast cancer and screening but were less familiar with screening-related policies. This might be because a high percentage of the participants were older than 40 years old (64.8%); thus, most had participated in the screening program (63.5% *vs*. 19.1%) and had more related knowledge (40.5% *vs*. 4.3%) by health departments compared with those younger than 40 years.

### 4.3 Strengths and limitations

This study has several advantages. First, to the best of our knowledge, this study is the first to develop a questionnaire to evaluate the KAP toward breast cancer screening among women working in the financial industry in Taiwan. Second, the present study identified several possible factors related to the practice of breast cancer screening, which could be valuable tools for developing interventions to increase participation rates. Our study also highlighted key practical barriers such as lack of time, limited access, and concerns about pain, which should be considered when developing and implementing these interventions.

This study has several limitations. First, the reliance on self-reported data for certain aspects of the study may have been influenced by a recall bias. Second, this study was not able to clarify the factors influencing practices of participating in the regular mammography. Future research should include measures of screening frequency to better examine predictive validity and long-term adherence behaviors. Third, although a factor analysis is a powerful tool for certain types of data, it was not suitable for our knowledge and practice scales because of the specific characteristics of these items. Fourth, the results regarding the internal consistency of the knowledge scales may not consistently measure a single underlying construct based on multiple objectives. To increase content validity (to incorporate all or most aspects of the phenomenon under study), the knowledge scale covers multiple subdomains, each representing a different concept with a relatively small number of items within. This design limitation contributed to low Cronbach’s alpha values for internal consistency. Additionally, while efforts were made to remove poorly performing items, the diversity of the subdomains and the weak correlations between them prevented significant improvements in reliability. Another limitation was the potential participant burden. Adding more items within each subdomain would likely increase the cognitive load on the participants, leading to potential fatigue and reduced data quality. As such, the scale could not comprehensively capture all subdomains in a manner that would enhance overall internal consistency while keeping the participant burden manageable. Future research should consider expanding the number of items within each subdomain and exploring alternative measurement strategies to improve psychometric reliability. Fifth, limitations regarding the psychometric properties of the knowledge and practice scales must be acknowledged, including the potential inadequacy of the factor analysis for these measures and the influence of self-report bias on responses. Sixth, we did not include women without paid work, which lost the potential to develop sensitive scales for female workers. Finally, although this study offers valuable insights into breast cancer screening attitudes and practices within a specific occupational group in Taiwan, the findings may have limited generalizability to other populations or countries due to differences in cultural norms, healthcare systems, and industry-specific contexts. However, the structure and content of the questionnaire may still be relevant in similar professional or institutional environments, particularly among female workers in the health-related, industrial, and corporate sectors with organized health promotion programs. Future research could explore the cross-cultural adaptability of the tool by validating and modifying it for use in other regions or occupational groups, considering local beliefs, language, and healthcare accessibility.

In conclusion, this study indicated that the Chinese version of the questionnaire developed to evaluate the attitudes toward and practices of breast cancer screening among female workers in the financial industry in Taiwan had good construct validation and internal consistency. Individuals with positive attitudes were more likely to participate in mammography. Therefore, promoting breast cancer screening should focus not only on improving knowledge and supportive policies but also on reducing individuals’ reluctance to participate in breast cancer screening. These practical barriers include a lack of time, limited access, and concerns about pain. Effective interventions should be both informative and easily accessible to encourage greater participation in mammography screening.

## Data Availability

The raw data supporting the conclusions of this article will be made available by the authors, without undue reservation.
