# Supplementary material for: Development and validation of a Chinese version of a questionnaire to evaluate the knowledge, attitude, and practice about breast cancer screening among financial female workers in Taiwan
Source: Front Oncol. 2025 Jun 26;15:1559622. doi: 10.3389/fonc.2025.1559622 (PMC12241044; doi:10.3389/fonc.2025.1559622)
Supplement: Supplementary file 1 [file DataSheet1.docx]

**SUPPLEMENTAL MATERIAL**

**Original Article**

**Development and validation of a Chinese version of a questionnaire to evaluate the knowledge, attitude, and practice about breast cancer screening among financial female workers in Taiwan**

Jia-Yi Lin PhD^a,b^, Ching-I Hung MS^c,d^, Tsai-Chung Li PhD^a,*^, Ta-Yuan Chang PhD^b,*^

^a^ Department of Public Health, College of Public Health, China Medical University, Taichung, Taiwan

^b^ Department of Occupational Safety and Health, College of Public Health, China Medical University, Taichung, Taiwan

^c^ Institute of Labor, Occupational Safety and Health, Ministry of Labor, New Taipei, Taiwan

^d^ Institute of Environmental and Occupational Health Sciences, National Taiwan University, Taipei, Taiwan

**Footnotes:**

^*^Corresponding authors: Prof. Tsai-Chung Li, Department of Public Health, College of Public Health, China Medical University; Prof. Ta-Yuan Chang, Department of Occupational Safety and Health, College of Public Health, China Medical University, 100 Sec.1 Jingmao Road, Taichung 406040, Taiwan. E-mail: tcli@mail.cmu.edu.tw; [tychang@mail.cmu.edu.tw](mailto:tychang@mail.cmu.edu.tw)

Telephone: 886-4-22053366 ext 6605 and 6203 Fax: 886-4-22079225.

Figure of contents: Page

[Figure S 1. The steps for developing the questionnaire on the knowledge, attitudes, and practice toward breast cancer screening. 4](#_Toc182480314)

Table of contents: Page

[Table S 1. Content validity of basic information by experts for original version questionnaire. 5](#_Toc182480629)

[Table S 2. Content validity of breast cancer knowledge by experts for original version questionnaire. 6](#_Toc182480630)

[Table S 3. Content validity of breast cancer attitude by experts for original version questionnaire. 7](#_Toc182480631)

[Table S 4. Content validity of breast cancer practice by experts for original version questionnaire. 8](#_Toc182480632)

[Table S 5. Distributions of items on breast cancer screening knowledge. (N=1,511). 9](#_Toc182480633)

[Table S 6. Distributions of items on breast cancer screening attitude. (N=1,511). 10](#_Toc182480634)

[Table S 7. The description on breast cancer screening practice. (N=1,511). 12](#_Toc182480635)

[Table S 8. The correlation between knowledge and attitude of breast cancer screening (N=1,511) 13](#_Toc182480636)

[Table S 9. Reliability test on knowledge and practice of breast cancer. (N=64). 14](#_Toc182480637)

[Table S 10. Differential analysis of the practice of ever participating in breast cancer screening. (N=1,511). 15](#_Toc182480638)

[Table S 11. The associations between sociodemographic factors and the practice of ever participating in the mammogram (N=1,511). 18](#_Toc182480639)

[Table S 12. The associations between sociodemographic factors and the practice of ever doing self-check. (N=1,511). 19](#_Toc182480640)

[Table S 13. The associations between sociodemographic factors and the practice of ever participating in the ultrasound examination. (N=1,511). 20](#_Toc182480641)

Figure S 1. The steps for developing the questionnaire on the knowledge, attitudes, and practice toward breast cancer screening.


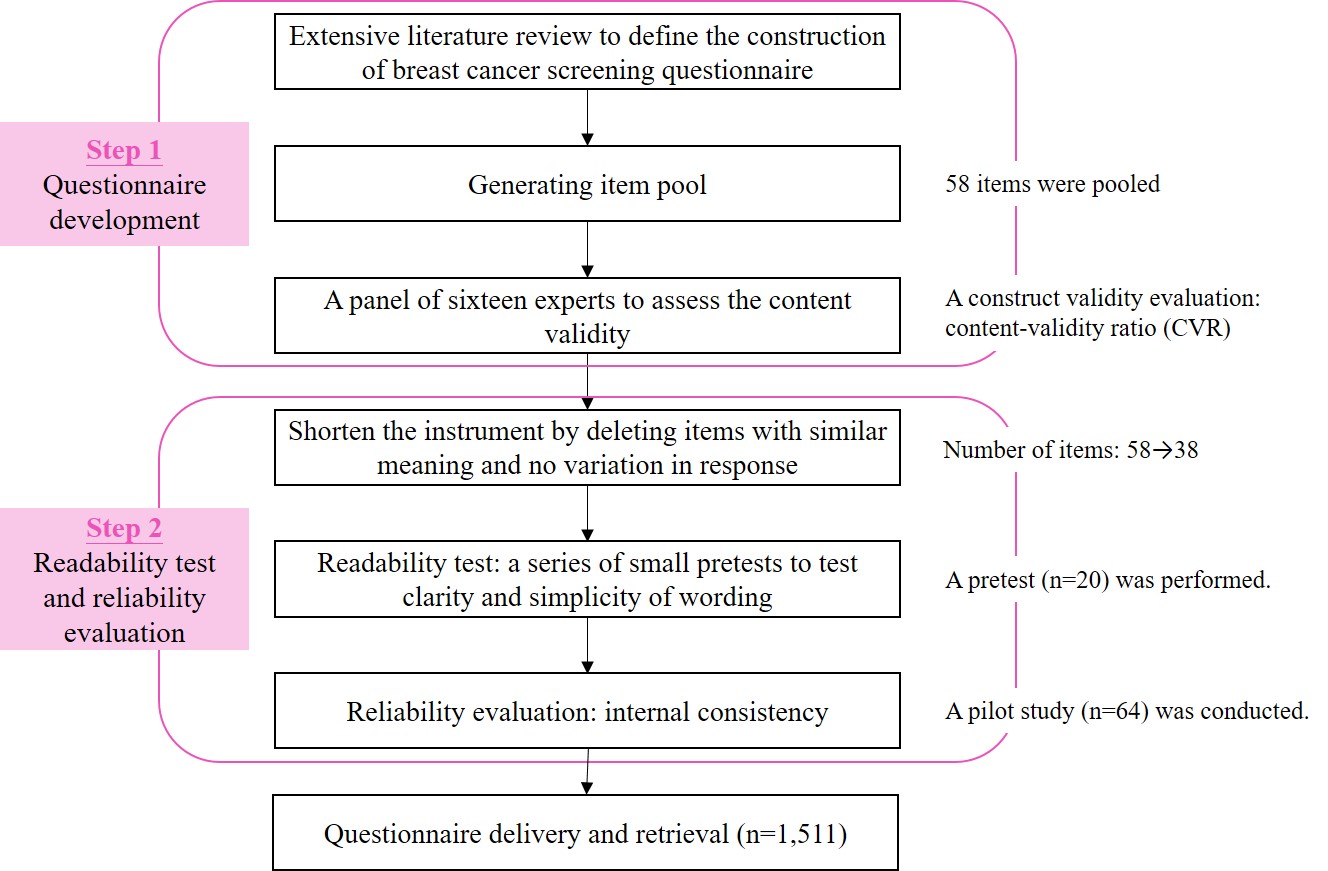


Table S 1. Content validity of basic information by experts for original version questionnaire.

| Basic information | CVR |
| --- | --- |
| *1. Your birth date: / (yyyy/mm)* | 1 |
| *2. Your education level:*  *(1) Junior high school education (2) Senior high school education*  *(3) College or University (4) Master degree or above* | 1 |
| *3. Your total monthly household income is:*  *(1) <50,000 NTD (2) 50,001-100,000 NTD*  *(3) 100,001-150,000 NTD (4) >150,000 NTD* | 1 |
| *4. Your recent marital status:*  *(1) Single (2) Married (3) Divorced (4) Widowed* | 1 |
| *5. Are you a current or former smoker? (At least 1 cigarette per day for at least 6 months)*  *(1) No (2) Yes, I am a current smoker (3) Yes, I am a former smoker* | 1 |
| *6. Do you have alcohol consumption currently or in the past? (Up to 150c.c. per week for at least 6 months)*  *(1) No (2) Yes, I have currently (3) Yes, I have in the past* | 1 |
| *7. Your first menstrual age: years old* | 1 |
| *8. Do you currently menstruating? (1) Yes (2) No, I stopped at: years old* | 1 |
| *9. Have you ever given birth to a child? (1) Never (2) Ever had* | 1 |
| *10. The number of your birth to a child: births* | 0.78 |
| *11. Your age at first given birth to a child: years old* | 0.78 |
| *12. Have you breastfed yourself with at least 1 child? (1) No (2) Yes* | 1 |
| *13. Have you ever taken birth control pills regularly (at least once a month for at least 6 months)?*  *(1) No (2) Yes* | 1 |
| *14. Have you ever been diagnosed with breast cancer by physicians?*  *(1) No (2) Yes, time of diagnosis: year (yyyy)* | 1 |
| *15. To the best of your knowledge, has any of your female relatives ever had breast cancer?*  *(1) No (2) Yes* | 1 |
| *16. Which female relatives of you have had breast cancer? (multiple choice)*  *(1) Grandmother (2) Mother (3) Aunt (4)Sister* | 1 |
| *17. Your working type:*  *(1) Day shift (2) Night shift (3) Shifting (4) Part time* | 1 |
| *18. Do you feel that your work pressure in the past month has caused troubles in your life?*  *(1) Never (2) Rarely (3) Sometimes (4) Often (5) Always* | 0.78 |
| *19. Are you satisfied with your current job?*  *(1) Dissatisfied (2) Not very satisfied (3) Normality satisfied*  *(4) Satisfied (5) Very satisfied* | 0.56 |

Table S 2. Content validity of breast cancer knowledge by experts for original version questionnaire.

| Knowledge of breast cancer | ○ | × | CVR |
| --- | --- | --- | --- |
| *1. Breast cancer at early stage is mostly a curable disease.* |  |  | 0.33 |
| *2. Nothing can change your fate of getting breast cancer.* |  |  | 0.78 |
| *3. Women who had ever breastfed are more likely to get breast cancer.* |  |  | 0.78 |
| *4. Women who had ever given birth are more likely to get breast cancer.* |  |  | 0.78 |
| *5. If one of your female relatives have had breast cancer, you are more likely to get breast cancer.* |  |  | 0.78 |
| *6. Women who have gone through menopause are more likely to get breast cancer.* |  |  | 0.78 |
| *7. People who take hormone drugs are more likely to get breast cancer.* |  |  | 0.78 |
| *8. Abnormal secretion of breast cancer is the clinical symptom of breast cancer.* |  |  | 0.78 |
| *9. Regular breast self-examination can achieve the purpose of early detection.* |  |  | 0.78 |
| *10. Going to the hospital for breast ultrasound examination is dangerous to your health.* |  |  | 0.78 |
| *11. Going to the hospital for a mammogram is dangerous to your health.* |  |  | 0.56 |
| *12. Going to the hospital for breast clinical examination is dangerous to your health.* |  |  | 0.78 |
| *13. I know that the National Health Service of the Ministry of Health and Welfare provides mammography every two years for women aged 45-69 years.* |  |  | 0.78 |
| *14. I know that the government provides free breast mammography once per three years for women.* |  |  | 0.56 |

Table S 3. Content validity of breast cancer attitude by experts for original version questionnaire.

| Attitude of breast cancer | 1 | 2 | 3 | 4 | 5 | CVR |
| --- | --- | --- | --- | --- | --- | --- |
| *1. It is not necessary for a mammogram when I think that my breasts are in good health.* |  |  |  |  |  | 0.78 |
| *2. It’s not necessary for a mammogram when I live with a healthy life (i.e. regular exercise, healthy diet).* |  |  |  |  |  | 0.78 |
| *3. It is save for me to have a mammogram.* |  |  |  |  |  | 0.78 |
| *4. It is necessary for me to have a mammogram.* |  |  |  |  |  | 0.78 |
| *5. Regular mammograms keep me on the health of my breasts.* |  |  |  |  |  | 0.78 |
| *6. Even if every breast self-exam is normal, I should keep getting mammograms.* |  |  |  |  |  | 0.78 |
| *7. I believe that participating in a mammogram will determine abnormalities early.* |  |  |  |  |  | 0.78 |
| *8. I am worried that having a mammogram will hurt my breasts.* |  |  |  |  |  | 0.75 |
| *9. I did not want to have a mammogram because my breasts would have to be unveiled.* |  |  |  |  |  | 1 |
| *10. Mammograms are painful, so I do not want to have mammograms.* |  |  |  |  |  | 0.75 |
| *11. I was too busy at work, so I do not want to have time for mammograms.* |  |  |  |  |  | 0.75 |
| *12. Lack of transportation would keep me from having a mammogram.* |  |  |  |  |  | 0.75 |
| *13. Having breast cancer would affect my life so I did not want to have a mammogram.* |  |  |  |  |  | 0 |
| *14. The cost burden is too high, so I do not want to have a mammogram.* |  |  |  |  |  | 0.5 |
| *15. There are no health facilities near my home to provide mammograms, so I do not want to have a mammogram.* |  |  |  |  |  | 0.75 |

1=strongly disagree, 2=disagree, 3=uncertain, 4=agree, 5=strongly agree.

Table S 4. Content validity of breast cancer practice by experts for original version questionnaire.

| Practice of breast cancer | CVR |
| --- | --- |
| *1. Have you had a breast self-exam within the past two years?*  *(0) No (1) Yes, but not regular (2) Yes, self-check regularly (at least 1 month)* | 1 |
| *2. Have you had a mammogram in the past two years? (0) No (1) Yes* | 1 |
| *3. How many mammograms have you had in the past two years?*  *(1) Once (2) Twice (3) Three times or more* | 1 |
| *4. Have you paid for a mammogram in the past two years?*  *(0) No (1) Yes* | 0.78 |
| *5. Have you had a breast ultrasound examinations within the past two years? (0) No (1) Yes* | 1 |
| *6. How many breast ultrasound examinations have you had in the past two years?*  *(1) Once (2) Twice (3) Three times (above)* | 1 |
| *7. Have you paid for a breast ultrasound examinations within the past two years?*  *(0) No (1) Yes* | 0.78 |
| *8. Has a doctor recommended you to be screened for breast cancer in the past two years?*  *(0) No (1) Yes* | 0.78 |
| *9. Have you ever received a notice from health agencies to recommend you for a breast cancer screening in the past two years?*  *(0) No (1) Yes* | 0.78 |
| *10. Have you been screened for breast cancer in the past two years?*  *(0) No (1) Yes* | 0.75 |

Table S 5. Distributions of items on breast cancer screening knowledge. (N=1,511).

| Items | Mean (SD) | Medium (Q1-Q3) |
| --- | --- | --- |
| Correct answer rate of breast cancer screening knowledge questions |  |  |
| BCK1: Breast cancer at early stage is mostly a curable disease. | 0.87 (0.33) |  |
| BCK2: Nothing can change your fate of getting breast cancer. | 0.83 (0.38) |  |
| BCK3: Women who had ever breastfed are more likely to get breast cancer. | 0.92 (0.27) |  |
| BCK4: Women who had ever given birth are more likely to get breast cancer. | 0.88 (0.32) |  |
| BCK5: If one of your female relatives have had breast cancer, you are more likely to get breast cancer. | 0.94 (0.24) |  |
| BCK6: Women who have gone through menopause are more likely to get breast cancer. | 0.85 (0.36) |  |
| BCK7: People who take hormone drugs are more likely to get breast cancer. | 0.67 (0.47) |  |
| BCK8: Abnormal secretion of breast cancer is the clinical symptom of breast cancer. | 0.92 (0.27) |  |
| BCK9: Regular breast self-examination can achieve the purpose of early detection. | 0.99 (0.11) |  |
| BCK10: Going to the hospital for breast ultrasound examination is dangerous to your health. | 0.96 (0.20) |  |
| BCK11: The benefit of having a mammogram is greater than its risk for your health. | 0.90 (0.30) |  |
| BCK12: Breast palpation by a physician is dangerous to your health. | 0.98 (0.14) |  |
| BCK13: The government provides free regular mammograms for women aged 45-65 years. | 0.05 (0.21) |  |
| BCK14: The government provides free regular mammogram examinations for women aged 40-44 years who have had breast cancer in their first-degree relatives. | 0.08 (0.28) |  |
| BCK15: The government provides free breast mammograms once per three years for women. | 0.24 (0.43) |  |
| Total score | 11.07 (1.45) | 11 (10-12) |

BCK1 to BCK15: questions on breast cancer screening knowledge from 1 to 15; SD: standard deviation.

Table S 6. Distributions of items on breast cancer screening attitude. (N=1,511).

| Items | Mean (SD) | Median (Q1-Q3) |
| --- | --- | --- |
| Attitude on doing a mammography |  |  |
| BCA1: A mammogram is not necessary if I believe my breasts are healthy. | 4.07 (0.94)^a^ |  |
| BCA2: A mammogram is not necessary because I live a healthy life (i.e., regular exercise, healthy diet). | 4.14 (0.85)^a^ |  |
| BCA3: I have the ability to check up my breasts, so I do not need to participate in the mammogram. | 4.21 (0.80)^a^ |  |
| BCA4: It is save for me to have a mammogram. | 4.14 (0.69) |  |
| BCA5: It is necessary for me to have a mammogram. | 4.17 (0.70) |  |
| BCA6: Regular mammograms keep me updated on the health of my breasts. | 4.30 (0.62) |  |
| BCA7: Regular mammograms provide medical information about breast cancer. | 4.24 (0.67) |  |
| BCA8: Even if every breast self-exam is normal, I should keep getting mammograms. | 4.21 (0.70) |  |
| BCA9: I believe that participating in a mammogram will determine abnormalities early. | 4.34 (0.62) |  |
| Total score | 37.78 (5.11) | 36 (36-42) |
| Attitude on the reasons for not participating in mammogram |  |  |
| BCA10: I am worried that having a mammogram will hurt my breasts. | 4.05 (0.81)^a^ |  |
| BCA11: I was uncomfortable with the idea of exposing my breasts during the mammogram. | 4.02 (0.92)^a^ |  |
| BCA12: Mammograms are painful, so I do not want to have them. | 3.82 (1.08)^a^ |  |
| BCA13: I am too busy at work, so I do not have time for mammograms. | 3.38 (1.18)^a^ |  |
| BCA14: Lack of transportation would keep me from having a mammogram. | 3.63 (1.12)^a^ |  |
| BCA15: Having breast cancer would affect my life, so I do not want to have a mammogram. | 4.27 (0.73)^a^ |  |
| BCA16: The cost is too high, which discourages me from getting a mammogram. | 3.96 (0.95)^a^ |  |
| BCA17: There are no health facilities near my home to provide mammograms, so I do not want to have a mammogram. | 3.88 (0.99)^a^ |  |
| Total score | 30.98 (5.68) | 31 (27-35) |

BCA1 to BCA17: questions on breast cancer screening attitude from 1 to 17; SD: standard deviation; ^a^ Reverse question turns to forward integral.

Table S 7. The description on breast cancer screening practice. (N=1,511).

| Breast cancer screening | N (%) | Age <40 | Age ≥40 |
| --- | --- | --- | --- |
| Self-check |  |  |  |
| Ever done breast self-check | **1,510 (99.9)** | **486 (32.2)** | **1, 024 (67.8)** |
| Never | 357 (23.6) | 206 (42.4) | 151 (14.7) |
| Ever | 1,153 (76.4) | 280 (57.6) | 873 (85.3) |
| Ultrasound examination |  |  |  |
| Ever done ultrasound examination | **1,508 (99.8)** | **486 (32.2)** | **1, 022 (67.8)** |
| Never | 470 (31.2) | 289 (59.5) | 181 (17.7) |
| Ever | 1,038 (68.8) | 197 (40.5) | 841 (82.3) |
| X-ray mammography |  |  |  |
| Ever done breast mammography | **1,484 (98.2)** | **482 (32.5)** | **1,002 (67.5)** |
| Never | 756 (50.9) | 390 (80.9) | 366 (36.5) |
| Ever | 728 (49.1) | 92 (19.1) | 636 (63.5) |
| Ever announced by health department to participate in the X-ray mammography | **318 (43.9)** | **4 (4.3)** | **259 (40.5)** |

Table S 8. The correlation between knowledge and attitude of breast cancer screening (N=1,511)

| Cronbach’s α | **Knowledge** | Knowledge on breast cancer | Knowledge on breast cancer screening | **Attitude** | Attitude on doing a mammography | Attitude on the reasons for not participating in mammogram |
| --- | --- | --- | --- | --- | --- | --- |
| **Knowledge** | (0.37) |  |  |  |  |  |
| Knowledge on breast  cancer | 0.84*** | (0.25) |  |  |  |  |
| Knowledge on breast  cancer screening | 0.68*** | 0.16*** | (0.35) |  |  |  |
| **Attitude** | 0.14*** | 0.10*** | 0.11*** | (0.91) |  |  |
| Attitude on doing a  mammography | 0.15*** | 0.12*** | 0.10* | 0.86*** | (0.91) |  |
| Attitude on the reasons for  not participating in  mammogram | 0.10* | 0.05* | 0.10*** | 0.89*** | 0.54*** | (0.87) |

*p<0.05; **p<0.01; ***p<0.001; Value in brackets is Cronbach’s α coefficient.

Table S 9. Reliability test on knowledge and practice of breast cancer. (N=64).

| Items | Kappa | 95% CI |
| --- | --- | --- |
| Knowledge | 0.14-0.72 (9/15) | − |
| Breast cancer | 0.14−0.62 (6/8) | − |
| BCK1 | 0.41 | 0.16-0.66 |
| BCK2 | 0.62 | 0.32-0.93 |
| BCK3 | 0.52 | 0.18-0.86 |
| BCK4 | 0.49 | 0.20-0.78 |
| BCK5 | 0.40 | 0.04-0.76 |
| BCK6 | 0.14 | -0.20-0.48 |
| BCK7 | 0.48 | 0.26-0.70 |
| BCK8 | 0.28 | -0.18-0.93 |
| Breast cancer screening | 0.17−0.72 (3/7) | − |
| BCK9 | 0.39 | -0.39-1.00 |
| BCK10 | 0.17 | -0.20-0.54 |
| BCK11 | 0.30 | -0.08-0.69 |
| BCK12 | 0.49 | -0.11-1.00 |
| BCK13 | 0.38 | -0.18-0.93 |
| BCK14 | 0.55 | 0.09-1.00 |
| BCK15 | 0.72 | 0.48-0.95 |
| Practice | 0.44−0.73 (3/3) | − |
| Self-check | 0.44 | 0.21-0.66 |
| Ultrasound examination | 0.73 | 0.56-0.91 |
| Mammography | 0.69 | 0.49-0.89 |

CI: confidence interval.

BCK1 to BCK15: questions on breast cancer knowledge from 1 to 15.

Numbers in brackets is questions with Kappa>0.40.

Table S 10. Differential analysis of the practice of ever participating in breast cancer screening. (N=1,511).

| Variables | Practice of breast cancer screening | | | | | |
| --- | --- | --- | --- | --- | --- | --- |
|  | Self-check  n (%) | *p-value* | Ultrasound  n (%) | *p-value* | Mammography  n (%) | *p-value* |
| Age (years) | **n=1153** | <0.001^a^ | **n=1038** | <0.001^a^ | **n=728** | <0.001^a^ |
| <40 | 316 (27.4) |  | 226 (21.8) |  | 119 (16.4) |  |
| ≥40 | 837 (72.6) |  | 812 (78.2) |  | 609 (83.7) |  |
| Living area | **n=1141** | <0.001^a^ | **n=1026** | <0.001^a^ | **n=719** | <0.001^a^ |
| Northern | 660 (57.8) |  | 621 (60.5) |  | 446 (62.0) |  |
| Western | 209 (18.3) |  | 159 (15.5) |  | 116 (16.1) |  |
| Southern | 258 (22.6) |  | 233 (22.7) |  | 150 (20.9) |  |
| Eastern | 14 (1.2) |  | 13 (1.3) |  | 7 (1.0) |  |
| Education level | **n=1149** | 0.492^a^ | **n=1036** | 0.239^a^ | **n=725** | 0.001^a^ |
| Senior high school education or below | 83 (7.2) |  | 77 (7.4) |  | 67 (9.2) |  |
| College or University or above | 1,066 (92.8) |  | 959 (92.6) |  | 658 (90.8) |  |
| Average monthly household income (Taiwan dollar) | **n=1144** | 0.002^a^ | **n=1029** | <0.001^a^ | **n=722** | <0.001^a^ |
| <100 thousand | 693 (60.6) |  | 611 (59.4) |  | 416 (57.6) |  |
| ≥100 thousand | 451 (39.4) |  | 418 (40.6) |  | 306 (42.4) |  |
| Marital status | **n=1151** | <0.001^a^ | **n=1036** | <0.001^a^ | **n=727** | <0.001^a^ |
| Married | 797 (69.2) |  | 753 (73.3) |  | 546 (75.1) |  |
| Single/ Divorced/ Widowed | 354 (30.8) |  | 277 (26.7) |  | 181 (24.9) |  |
| Cigarette smoking | **n=1152** | 1.000^a^ | **n=1037** | 0.710^a^ | **n=728** | 0.726^a^ |
| Current | 6 (0.5) |  | 5 (0.5) |  | 3 (0.4) |  |
| Never or former | 1,146 (99.5) |  | 1,032 (99.5) |  | 725 (99.6) |  |
| Alcohol consumption | **n=1151** | 0.262^a^ | **n=1036** | 0.556^a^ | **n=726** | 0.372^a^ |
| Current | 54 (4.7) |  | 50 (4.8) |  | 33 (4.5) |  |
| Never or former | 1,097 (95.3) |  | 986 (95.2) |  | 693 (95.5) |  |
| Size of institutions | **n=1153** | 0.381^a^ | **n=1038** | <0.001^a^ | **n=728** | <0.001^a^ |
| Small | 314 (27.2) |  | 309 (29.8) |  | 230 (31.6) |  |
| Medium | 155 (13.4) |  | 133 (12.8) |  | 96 (13.2) |  |
| Large | 684 (59.3) |  | 596 (57.4) |  | 402 (55.2) |  |
| Type of position | **n=1151** | <0.001^a^ | **n=1036** | <0.001^a^ | **n=727** | <0.001^a^ |
| Clerk | 658 (57.2) |  | 567 (54.7) |  | 353 (48.6) |  |
| Supervisor | 235 (20.4) |  | 235 (22.7) |  | 198 (27.2) |  |
| Others | 258 (22.4) |  | 234 (22.6) |  | 176 (24.2) |  |
| Current menstrual status | **n=1153** | <0.001^a^ | **n=1038** | <0.001^a^ | **n=728** | <0.001^a^ |
| Yes | 871 (75.5) |  | 745 (71.8) |  | 459 (63.0) |  |
| No | 282 (24.5) |  | 293 (28.2) |  | 269 (37.0) |  |
| Diagnosed with breast cancer by a physician | **n=1150** | 0.264^a^ | **n=1036** | 0.007^a^ | **n=725** | <0.001^a^ |
| Yes | 19 (1.7) |  | 21 (2.0) |  | 19 (2.6) |  |
| No | 1,131 (98.3) |  | 1,015 (98.0) |  | 706 (97.4) |  |
| Female relative with breast cancer | **n=1152** | 0.416^a^ | **n=1037** | 0.020^a^ | **n=728** | 0.003^a^ |
| Yes | 206 (17.9) |  | 197 (19.0) |  | 146 (20.1) |  |
| No | 946 (82.1) |  | 840 (81.0) |  | 582 (79.9) |  |
| Knowledge | **n=1153** | <0.001^a^ | **n=1038** | <0.001^a^ | **n=728** | <0.001^a^ |
| Q1 (<10 score) | 120 (10.4) |  | 105 (10.1) |  | 71 (9.8) |  |
| Q2 (10~11 score) | 184 (16.0) |  | 158 (15.2) |  | 116 (15.9) |  |
| Q3 (11~12 score) | 341 (29.6) |  | 305 (29.4) |  | 197 (27.1) |  |
| Q4 (≥12 score) | 508 (44.0) |  | 470 (45.3) |  | 344 (47.2) |  |
| Attitude |  |  |  |  |  |  |
| Total score of breast cancer attitudes toward receiving a mammography | **n=1153** | <0.001^a^ | **n=1038** | <0.001^a^ | **n=728** | <0.001^a^ |
| Q1 (<36 score) | 259 (22.5) |  | 208 (20.0) |  | 127 (17.4) |  |
| Q2 (36 score) | 304 (26.4) |  | 269 (25.9) |  | 188 (25.8) |  |
| Q3 (36~42 score) | 257 (22.3) |  | 245 (23.6) |  | 161 (22.1) |  |
| Q4 (≥42 score) | 333 (28.9) |  | 316 (30.4) |  | 252 (34.6) |  |
| Total score of breast cancer attitudes toward the reasons for not receiving a mammography | **n=1153** | <0.001^a^ | **n=1038** | <0.001^a^ | **n=728** | <0.001^a^ |
| Q1 (<27 score) | 243 (21.1) |  | 192 (18.5) |  | 111 (15.2) |  |
| Q2 (27~31 score) | 242 (21.0) |  | 212 (20.4) |  | 135 (18.5) |  |
| Q3 (31~35 score) | 338 (29.3) |  | 320 (30.8) |  | 232 (31.9) |  |
| Q4 (≥35 score) | 330 (28.6) |  | 314 (30.3) |  | 250 (34.3) |  |

^a^ Chi-square test.

Table S 11. The associations between sociodemographic factors and the practice of ever participating in the mammogram (N=1,511).

| Variables | Mammography | | | | | |
| --- | --- | --- | --- | --- | --- | --- |
|  | OR | 95% CI | *p-value* | AOR | 95% CI | *p-value* |
| Age (years), ≥40 vs. <40 (ref.) | 7.37 | 5.67−9.56 | <0.001 | 3.41 | 2.52-4.64 | <0.001 |
| Living area, western vs. northern (ref.) | 0.59 | 0.46−0.77 | <0.001 | 0.68 | 0.49-0.95 | 0.022 |
| Living area, southern vs. northern (ref.) | 0.86 | 0.67−1.09 | 0.214 | 0.59 | 0.42-0.81 | 0.001 |
| Living area, eastern vs. northern (ref.) | 0.91 | 0.33−2.52 | 0.853 | 0.61 | 0.19-1.94 | 0.407 |
| Education level, College or University or above vs. below senior high (ref.) | 0.51 | 0.33−0.77 | 0.001 | − | − | − |
| Average monthly household income (Taiwan Dollar), ≥100 thousand vs. <100 thousand | 1.54 | 1.24-1.90 | <0.001 | − | − | − |
| Marital status, single/ divorced/ widowed vs. married (ref.) | 0.36 | 0.29−0.45 | <0.001 | 0.72 | 0.55-0.95 | 0.020 |
| Cigarette smoking, current vs. never or former (ref.) | 0.62 | 0.15−2.61 | 0.515 | − | − | − |
| Alcohol consumption, current vs. never or former (ref.) | 0.81 | 0.51−1.29 | 0.373 | − | − | − |
| Size of institutions, medium vs. large (ref.) | 0.95 | 0.71−1.28 | 0.748 | 1.31 | 0.86-2.01 | 0.212 |
| Size of institutions, small vs. large (ref.) | 0.64 | 0.52−0.78 | <0.001 | 0.74 | 0.54-1.02 | 0.066 |
| Type of position, supervisor vs. clerk (ref.) | 3.50 | 2.61−4.68 | <0.001 | 2.11 | 1.48-3.00 | <0.001 |
| Type of position, others vs. clerk (ref.) | 1.32 | 1.03−1.69 | 0.028 | 1.45 | 1.07-1.97 | 0.016 |
| Current menstrual status, yes vs. no (ref.) | 0.13 | 0.10−0.18 | <0.001 | 0.22 | 0.16-0.32 | <0.001 |
| Diagnosed with breast cancer by a physician, yes vs. no (ref.) | 10.15 | 2.36−43.71 | 0.002 | − | − | − |
| Female relative with breast cancer, yes vs. no (ref.) | 1.50 | 1.14−1.97 | 0.004 | − | − | − |
| Total score of breast cancer knowledge, 1-IQR (2 points) | 0.91 | 0.69−1.20 | 0.514 | 1.12 | 0.94-1.34 | 0.211 |
| Total score of breast cancer attitudes toward receiving a mammography,  1-IQR (6 points) | 1.03 | 0.80−1.31 | 0.844 | 1.47 | 1.24-1.75 | <0.001 |
| Total score of breast cancer attitudes toward the reasons for not receiving a mammography, 1-IQR (8 points) | 2.67 | 2.08−3.41 | <0.001 | 1.79 | 1.46-2.20 | <0.001 |

OR: odds ratio; AOR: adjusted odds ratio.

Table S 12. The associations between sociodemographic factors and the practice of ever doing self-check. (N=1,511).

| Variables | Self-check | | | | | |
| --- | --- | --- | --- | --- | --- | --- |
|  | OR | 95% CI | *p-value* | AOR | 95% CI | *p-value* |
| Age (years), ≥40 vs. <40 (ref.) | 4.25 | 3.32−5.46 | <0.001 | 2.91 | 2.21−3.83 | <0.001 |
| Living area, western vs. northern (ref.) | 0.57 | 0.43−0.75 | <0.001 | − | − | − |
| Living area, southern vs. northern (ref.) | 1.18 | 0.88−1.59 | 0.268 | − | − | − |
| Living area, eastern vs. northern (ref.) | 4.38 | 0.57−33.39 | 0.1546 | − | − | − |
| Education level, College or University or above vs. below senior high (ref.) | 0.84 | 0.52−1.37 | 0.4921 | − | − | − |
| Average monthly household income (Taiwan dollar), ≥100 thousand vs. <100 thousand | 1.51 | 1.17−1.95 | 0.0016 | − | − | − |
| Marital status, single/ divorced/ widowed vs. married (ref.) | 0.36 | 0.28−0.46 | <0.001 | 0.62 | 0.47−0.81 | <0.001 |
| Cigarette smoking, current vs. never or former (ref.) | 0.93 | 0.19−4.62 | 0.9273 | − | − | − |
| Alcohol consumption, current vs. never or former (ref.) | 0.75 | 0.45−1.25 | 0.2635 | − | − | − |
| Size of institutions, medium vs. large (ref.) | 0.95 | 0.68−1.34 | 0.7844 | − | − | − |
| Size of institutions, small vs. large (ref.) | 0.88 | 0.69−1.12 | 0.2898 | − | − | − |
| Type of position, supervisor vs. clerk (ref.) | 1.97 | 1.38−2.82 | 0.0002 | − | − | − |
| Type of position, others vs. clerk (ref.) | 1.18 | 0.88−1.59 | 0.268 | − | − | − |
| Current menstrual status, yes vs. no (ref.) | 0.54 | 0.39−0.74 | 0.0002 | − | − | − |
| Diagnosed with breast cancer by a physician, yes vs. no (ref.) | 1.98 | 0.58−6.74 | 0.273 | − | − | − |
| Female relative with breast cancer, yes vs. no (ref.) | 1.14 | 0.83−1.57 | 0.4165 | − | − | − |
| Total score of breast cancer knowledge, 1-IQR (2 points) | 1.68 | 1.42−1.98 | <0.001 | 1.37 | 1.15−1.64 | <0.001 |
| Total score of breast cancer attitudes toward receiving a mammography, 1-IQR (6 points) | 1.29 | 1.13−1.48 | <0.001 | 1.05 | 0.88−1.24 | 0.611 |
| Total score of breast cancer attitudes toward the reasons for not receiving a mammography, 1-IQR (8 points) | 1.63 | 1.37−1.93 | <0.001 | 1.43 | 1.16−1.77 | <0.001 |

OR: odds ratio; AOR: adjusted odds ratio.

Table S 13. The associations between sociodemographic factors and the practice of ever participating in the ultrasound examination. (N=1,511).

| Variables | Ultrasound examination | | | | | |
| --- | --- | --- | --- | --- | --- | --- |
|  | OR | 95% CI | *p-value* | AOR | 95% CI | *p-value* |
| Age (years), ≥40 vs. <40 (ref.) | 6.82 | 5.35−8.68 | <0.001 | 4.22 | 3.15−5.65 | <0.001 |
| Living area, western vs. northern (ref.) | 0.39 | 0.30−0.50 | <0.001 | 0.47 | 0.34−0.66 | <0.001 |
| Living area, southern vs. northern (ref.) | 1.16 | 0.89−1.52 | 0.286 | 0.76 | 0.54−1.07 | 0.117 |
| Living area, eastern vs. northern (ref.) | 2.97 | 0.67−13.20 | 0.153 | 1.37 | 0.28−6.63 | 0.694 |
| Education level, College or University or above vs. below senior high (ref.) | 0.76 | 0.49−1.20 | 0.241 | 1.92 | 1.11−3.31 | 0.019 |
| Average monthly household income (Taiwan dollar), ≥100 thousand vs. <100 thousand | 1.62 | 1.28−2.05 | <0.001 | − | − | − |
| Marital status, single/ divorced/ widowed vs. married (ref.) | 0.27 | 0.21−0.33 | <0.001 | 0.48 | 0.37−0.64 | <0.001 |
| Cigarette smoking, current vs. never or former (ref.) | 0.75 | 0.18−3.17 | 0.700 | − | − | − |
| Alcohol consumption, current vs. never or former (ref.) | 0.86 | 0.53−1.41 | 0.556 | − | − | − |
| Size of institutions, medium vs. large (ref.) | 0.81 | 0.60−1.11 | 0.189 | − | − | − |
| Size of institutions, small vs. large (ref.) | 0.70 | 0.56−0.87 | 0.002 | − | − | − |
| Type of position, supervisor vs. clerk (ref.) | 3.15 | 2.21−4.49 | <0.001 | − | − | − |
| Type of position, others vs. clerk (ref.) | 1.18 | 0.90−1.55 | 0.229 | − | − | − |
| Current menstrual status, yes vs. no (ref.) | 0.24 | 0.17−0.34 | <0.001 | 0.45 | 0.29−0.68 | <0.001 |
| Diagnosed with breast cancer by a physician, yes vs. no (ref.) | 9.70 | 1.30−72.24 | 0.027 | − | − | − |
| Female relative with breast cancer, yes vs. no (ref.) | 1.43 | 1.06−1.94 | 0.020 | − | − | − |
| Total score of breast cancer knowledge, 1-IQR (2 points) | 1.66 | 1.43−1.94 | <0.001 | 1.16 | 0.97−1.39 | 0.106 |
| Total score of breast cancer attitudes toward receiving a mammography, 1-IQR (6 points) | 1.65 | 1.44−1.89 | <0.001 | 1.45 | 1.22−1.72 | <0.001 |
| Total score of breast cancer attitudes toward the reasons for not receiving a mammography, 1-IQR (8 points) | 2.01 | 1.70−2.37 | <0.001 | 1.62 | 1.31−2.01 | <0.001 |

OR: odds ratio; AOR: adjusted odds ratio.
